# One nanoprobe, two pathogens: gold nanoprobes multiplexing for point-of-care

**DOI:** 10.1186/s12951-015-0109-1

**Published:** 2015-08-07

**Authors:** Bruno Veigas, Pedro Pedrosa, Fábio F Carlos, Liliana Mancio-Silva, Ana Rita Grosso, Elvira Fortunato, Maria M Mota, Pedro V Baptista

**Affiliations:** Nanomedicine@FCT, Departamento de Ciências da Vida, Faculdade de Ciências e Tecnologia, CIGMH, UCIBIO, Universidade Nova de Lisboa, Campus de Caparica, 2829-516 Caparica, Portugal; Departamento de Ciência dos Materiais, Faculdade de Ciências e Tecnologia, CENIMAT/I3N, Universidade Nova de Lisboa, Caparica, Portugal; STABVIDA, Investigação e Serviços em Ciências Biológicas, Lda. Madan Parque, 2825-182 Caparica, Portugal; Instituto de Medicina Molecular, Universidade de Lisboa. Av. Prof. Egas Moniz, 1649-028 Lisbon, Portugal

**Keywords:** MTBC, Malaria, Nanodiagnostics, Gold nanoparticles, Tuberculosis, rpoB, Plasmodium

## Abstract

**Background:**

Gold nanoparticles have been widely employed for biosensing purposes with remarkable efficacy for DNA detection. Amongst the proposed systems, colorimetric strategies based on the remarkable optical properties have provided for simple yet effective sequence discrimination with potential for molecular diagnostics at point of need. These systems may also been used for parallel detection of several targets to provide additional information on diagnostics of pathogens.

**Results:**

For the first time, we demonstrate that a single Au-nanoprobe may provide for detection of two distinct targets (pathogens) allowing colorimetric multi-target detection. We demonstrate this concept by using one single gold-nanoprobe capable to detect members of the *Mycobacterium tuberculosis* complex and *Plasmodium sp*., the etiologic agents of tuberculosis and malaria, respectively. Following characterisation, the developed gold-nanoprobe allowed detection of either target in individual samples or in samples containing both DNA species with the same efficacy.

**Conclusions:**

Using one single probe via the non-cross-linking colorimetric methodology it is possible to identify multiple targets in one sample in one reaction. This proof-of-concept approach may easily be integrated into sensing platforms allowing for fast and simple multiplexing of Au-nanoprobe based detection at point-of-need.

**Electronic supplementary material:**

The online version of this article (doi:10.1186/s12951-015-0109-1) contains supplementary material, which is available to authorized users.

## Background

Most molecular diagnostic approaches for pathogen screening have been optimized for single pathogen identification, which is time consuming, costly and rely on highly trained technicians [1, 2]. Among these, particular emphasis has been brought upon molecular characterization based on PCR amplification and DNA hybridization approaches that detect specific molecular signatures in a few hours, which compare well to classical methods that can take up several days to yield a definite result [3, 4]. Despite being suitable for centralized well-equipped laboratories, these methodologies fail to deliver when moved to more remote locations and when a fast screening is required for assistance to clinical protocols. As such, the development of cheap, fast and user-friendly molecular methods at point-of-need is required and would have a huge impact in the capacity of early diagnosis and treatment of pathogen related infections. Fast and accurate results, i.e. early detection, are of utmost importance for the management of patients in terms of pathogen infection spreading and proper implementation of control and treatment measures [2]. Tuberculosis (TB) and malaria are two major global public health threats that undermine development in many resource-poor and some transitional settings with a substantial humanitarian, economic, and social impact [5, 6]. TB is still one of the leading human infectious diseases responsible for more than 1.5 million deaths and 9 million infections in 2013, together with the increasing rate of multidrug-resistant tuberculosis (MDRTB) pose a serious public health problem [7]. Malaria is endemic in 109 countries and was responsible for an estimated 207 million cases worldwide in 2012, with an estimated 627 000 deaths [6, 8, 9]. As observed with TB, the major burden of malaria occurs in sub-Saharan Africa and Asia. Human co-infection with these two pathogens is quite common [6–8] and represents an important public health problem in co-endemic areas of low and middle income countries [6].

Nanotechnology has already provided for powerful tools for molecular diagnostics. Numerous nanoparticle-based approaches have been designed to identify molecular pathogen signatures with extra sensitivity and faster than ever before [10–13]. Nowadays researchers have been gearing their efforts towards the development of nanotechnology-based systems that are affordable, robust and reproducible [14]. As such, nanoparticle based approaches are expected to evolve incrementally over time allowing to meet the needs faced in the field. In particular, systems based on gold nanoparticles (AuNPs) functionalized with thiol-modified DNA (Au-nanoprobes) have been extensively used for the detection and characterization of pathogens [10], including *Mycobacterium tuberculosis* [11, 12]. We have previously reported a detection strategy for members of the *M. tuberculosis* complex (MTBC) based on the observable colorimetric alteration of an Au-nanoprobe colloidal suspension [15–18]. The colorimetric alteration results from the differential aggregation profiles of Au-nanoprobes induced by increased ionic strength in the presence or absence of the specific target sequence: presence of the complementary target sequence prevents aggregation and the solution remains red (localized surface plasmon resonance (LSPR) band at 525 nm); whereas absence of a specific target sequence leads to nanoparticles aggregation after salt addition and the solution turns blue (red-shift of the LSPR peak to longer wavelength, 600–650 nm) [10]. This non-cross-linking method has shown to be extremely sensitive allowing for single point mismatches characterization, and was already applied to the identification of point mutations associated to rifampicin and isoniazid resistance in MTBC [12, 16–18].

**Fig. 1:**
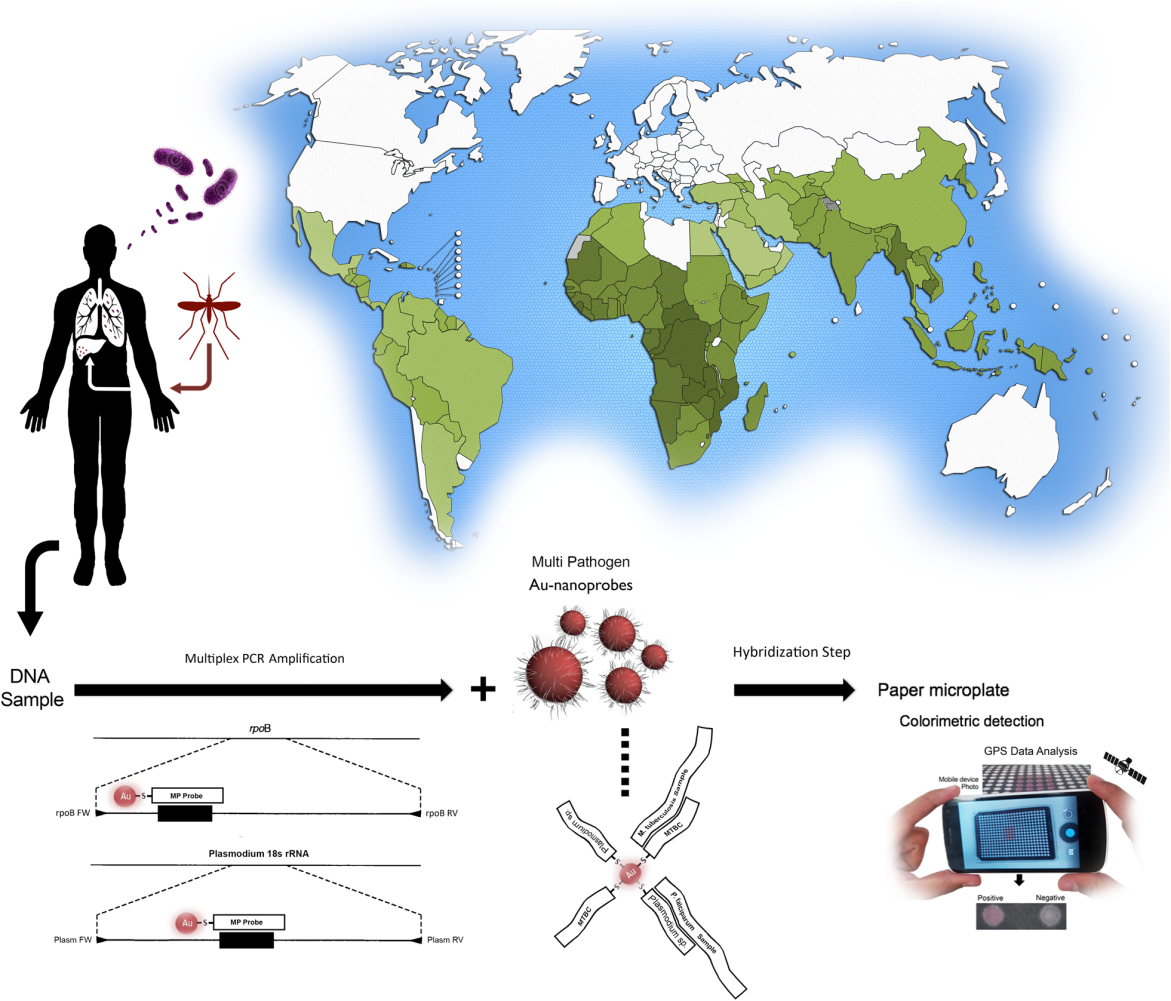
Multiplexing Au-nanoprobe based detection. DNA extracted from several biological fluids is amplified (e.g. PCR) and then a single Au-nanoprobe is used to detect presence of pathogens. Depicted is the detection of *M. tuberculosis* and *P. falciparum*. Map shows the geografical differences of Malaria and TB incidence where the highest rates are found predominantly in low-income countries in Africa and South America. This approach can be transposed into a simple and portable molecular diagnostic platform to be used at peripheral laboratories and/or point-of-need, coupled to a “smartphone” for data analysis and location metadata for real-time epidemiologic data [15].

Efforts have also been made to extend these system to allow for extra layers of information in a single reaction, either by exploring the differential kinetics of aggregation and hybridization efficiencies or relying on the use of additional plasmon signatures of NPs (e.g. gold:silver alloy nanoprobes) [18–21]. Here, we demonstrate that the Au-nanoprobe approach may be easily extended towards the simultaneous detection of two pathogens of interest with a single multi-sequence functionalized Au-nanoprobe to evaluate in a single test the presence/absence of any of the pathogens. To enhance the detection potential of this approach for MTBC and *Plasmodium* sp., we performed a preparative multiplex amplification of the respective specific *loci*—see Fig. 1. This concept extends the colorimetric detection to multiplexing suitable for point-of-need molecular screening.

## Methods

All reagents were purchased from Sigma Aldrich and were of analytical grade. HPLC purified labeled oligonucleotides were purchased from STABVIDA (Portugal) and used without further purification. Thiolated oligonucleotides were used to synthesize the Au-nanoprobes and non-modified oligonucleotides were used as specific controls for calibration of the assay.

### Biological samples

Clinical isolates obtained from respiratory samples positive for acid-fast bacilli (BAAR) from patients of the Lisbon Health Region were used as Tuberculosis positive control (*M. tuberculosis* H37Rv-ATCC27294^T^). *P. falciparum* 3D7 clone were used as Malaria positive control (*P. falciparum*–M19172.1).

### Multiplex PCR amplification

Two conserved regions of MTBC *rpoB* gene and *Plasmodium* 18 s ribosomal RNA (18 s rRNA) were amplified in a single and multiplex PCR reaction using two sets of primer pairs (Additional file 1: Table S1) designed to obtain two different fragments: 395 bp *rpoB* and 408 bp 18 s rRNA. PCR amplifications were performed on a Biometra^®^TGradient Thermocycler (Göttingen, Germany) in 50 µL final volume with 1× DreamTaq Buffer, 0.1 mM of each DNTPs, 2 µM of each primer and 0.1 U\µL of DreamTaq DNA polymerase (Amersham Biosciences, GE Healthcare, Europe) and ~1 µg/mL of template DNA with the following thermal cycling conditions: initial 5 min denaturation at 95°C, followed by 30 amplification cycles of denaturation at 95°C for 30 s, annealing at 58°C for 30 s, elongation at 72°C for 45 s, and a final elongation at 72°C for 5 min. PCR products were analyzed in a 1% agarose gel electrophoresis and quantified via UV/Vis spectroscopy (Nanodrop ND1000, Nanodrop Technologies, USA) (Additional file 1: Figure S1).

### Au-nanoprobes design and syntheses

Au-nanoprobes sequences were designed using Serial Cloner v. 1.3-11 and Geneious v. R7.1 comparative tools aligning the probe sequence with the targeted genes. The multi-functionalized Au-nanoprobe was designed for the detection of two pathogen with two specific sequences in a 1:80:80 (AuNP: oligo1: oligo2) ratio (Additional file 1: Table S1): one sequence complementary to MTBC members [14], and complementary to the 18S rRNA gene sequences from *Plasmodium sp*. For each of the used sequences a single functionalized Au-nanoprobe was also synthesized in a 1:160 (AuNP:oligo) ratio. Gold nanoparticles, with an average diameter of ~14 nm, were synthesized and functionalized as described by Veigas et al. [16] (Additional file 1: Figure S2). Briefly, thiol-modified oligonucleotides were incubated with the AuNPs with increasing salt concentration, in order to reduce non-specific bonds between the thiol-modified oligonucleotides and the AuNPs. After 16 h, the solution was centrifuged, the resulting pellet resuspended in 10 mM phosphate buffer (pH 8), 0.1 M NaCl, and stored in the dark at 4°C till further use.

### Au-nanoprobe colorimetric assay

The colorimetric assays were performed in a final volume of 30 μL containing Au-nanoprobes at a final concentration of 2.5 nM in 10 mM phosphate buffer (pH 8) and multiplex PCR product at final DNA concentration of 60 µg/mL. The mixture was heated up at 95°C for 5 min and then cooled down to 25°C for 5 min. For each probe, the assay consisted on the spectrophotometric comparison of a “Blank” (without DNA), 10 mM phosphate buffer (pH 8), 0.1 M NaCl; non-related control containing non-complementary DNA; and the samples. The pre-determined MgCl_2_ concentration was added to each reaction, and after 30 min at room temperature for color development, the mixtures and the blank assayed by UV/visible spectroscopy in a microplate reader (Tecan Infinite M200). For calibration, each set of Au-nanoprobes was tested against purified simplex PCR amplicons.

### Data analysis

Aggregation profiles were analyzed in terms of the ratio of Abs_525nm_/Abs_600nm_ (dispersed vs. aggregated species) for each Au-nanoprobe. Each probe was used in a minimum of three individual parallel hybridization experiments with the PCR amplified amplicons derived from the *rpoB* and 18 s rRNA genes from MTBC and *P. falciparum*, respectively. A threshold of 1 was considered where values >1 indicate that the Au-nanoprobe is mostly non-aggregated (Positive), whereas a value <1 indicate aggregation (Negative) [18]. This approach provides for indication of presence or absence of pathogens DNA in the sample.

## Results and discussion

Based on the molecular signatures of pathogens from MTBC and *Plasmodium* sp., we developed a two-step approach relying on a multiplex PCR amplification and subsequent hybridization with a single Au-nanoprobe. We targeted two *loci*: *rpoB* for MTBC and 18 s rRNA genomic sequences for *Plasmodium* sp. The specific set of primer allow amplification of unique genomic regions for strains belonging to these sub-groups and constitute the first line of selective detection, then the Au-nanoprobe assay a serves as the second line of identification (see Fig. 1). The presented method relies on the differential aggregation profile of Au-nanoprobes following an increase to ionic strength of the medium (e.g. salt addition, MgCl_2_). Hybridization to the complementary target stabilizes the Au-nanoprobe from the induced aggregation and the solution remains red; whereas no hybridization (no complementary target) leads to extensive aggregation and concomitant red-shift of the plasmon band and the solution turns blue [18]. The two *loci* were selected to allow for discriminatory detection of human pathogens: *rpoB* primers and MTBC probe specifically recognize members of the *M. Tuberculosis* complex; detection of 18 s rRNA genomic sequences for the most common species of parasites, *P. Falciparum*, *P. Vivax*, *P. malariae*, and *P. Ovale* [8]. The latter is also capable to recognize other more uncommon species, such as *P. knowlesi* and primate infecting type *Plasmodium cynomolgi*, with a recent reported case of natural human infection [22]. This screening approach assures that any of the species capable of infecting humans are amplified and detected by the nanoprobe assay.

**Fig. 2:**
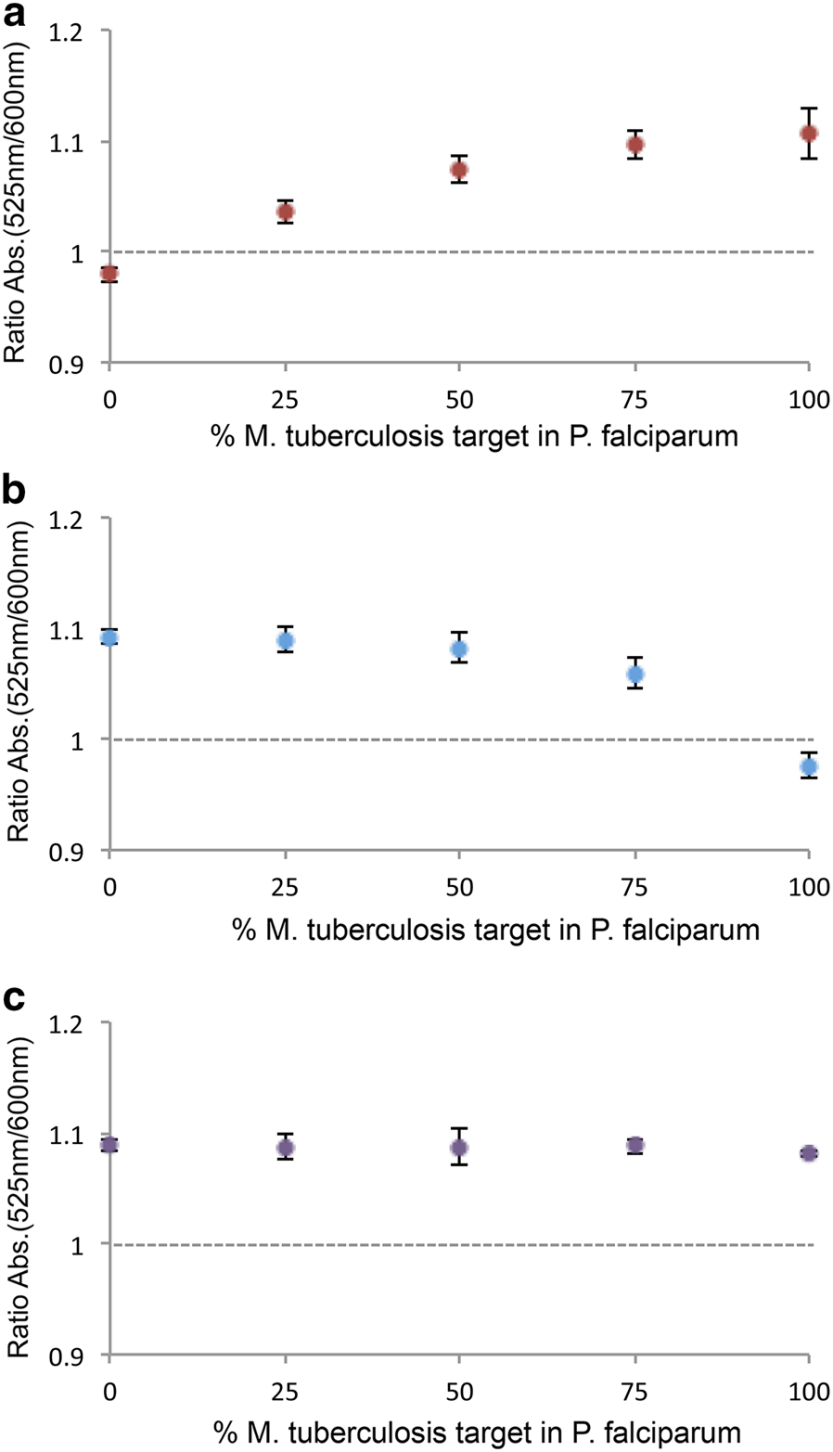
Single and multiple pathogen Au-nanoprobe specificity analysis. **a** MTBC probe; **b** *Plasmodium* *sp.* probe; **c** Multiple pathogen Au-nanoprobe. Assay performed in a microplate reader. Au-nanoprobe aggregation as measured by ratio of aggregation (ratio of SPR intensity at 525 and 600 nm) for the assay mixtures—2.5 nM Au-nanoprobe, 10 mM phosphate buffer (pH 8) + 0.1 M NaCl, and different percentages of each PCR amplified amplicon maintaining the final concentration of DNA at 60 ng/µL. All spectrophotometric data was collected 30 min after salt addition and *error bars* represent the standard deviation of three independent assays. The *horizontal line* represents the threshold of 1 considered for discrimination between positive (rAbs ≥ 1) and negative (rAbs < 1) result. A representative colorimetric results is showed upon each result *bar-red* positive result; *blue*/*purple* negative result.

**Fig. 3:**
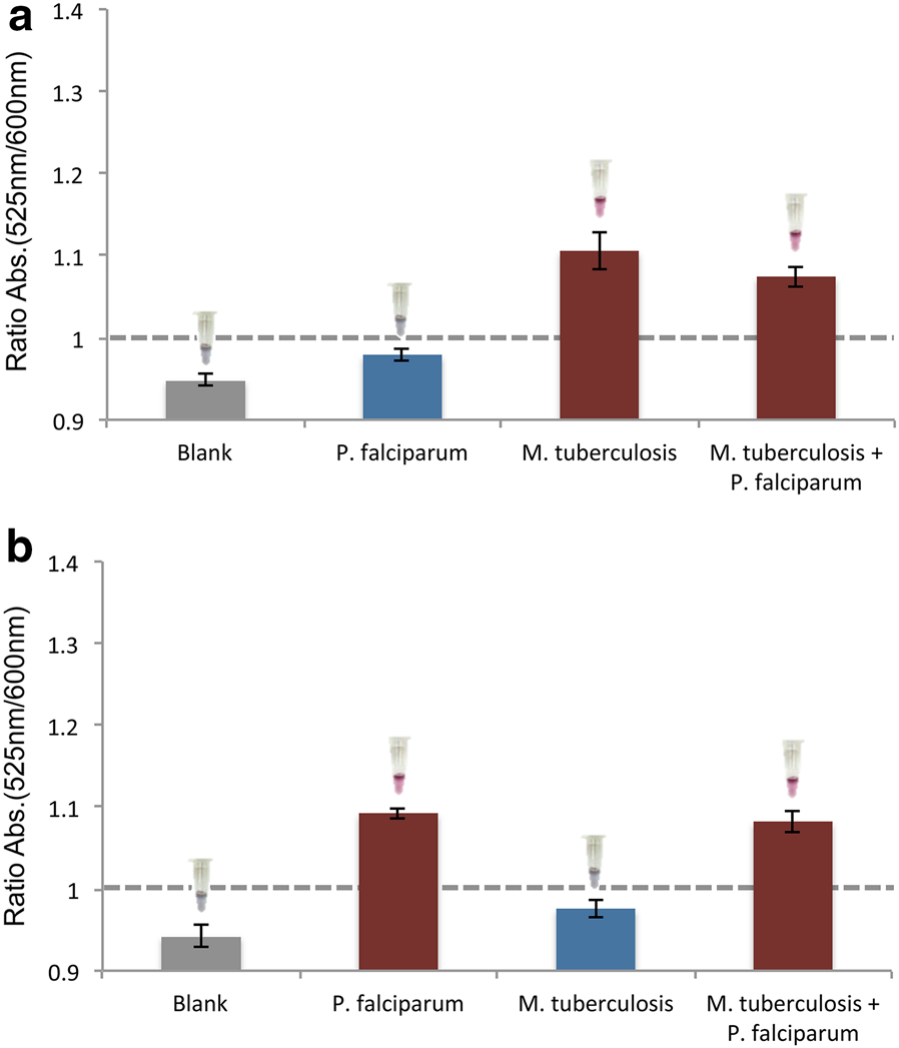
Single pathogen Au-nanoprobe detection assays using **a** MTBC probe; **b** *Plasmodium* sp. probe. The colorimetric assay consists of visual comparison of test solutions after salt induced Au-nanoprobe aggregation. Au-nanoprobes aggregation was measured by ratio of aggregation (ratio of SPR intensity at 525 and 600 nm) for the assay mixtures—2.5 nM Au-nanoprobe, 10 mM phosphate buffer (pH 8) + 0.1 M NaCl, purified dsDNA targets (PCR products) at a final concentration of 60 ng/µL. All spectrophotometric data was collected 30 min after salt addition and *error bars* represent the standard deviation of three independent assays. The *horizontal line* represents the threshold of 1 considered for discrimination between positive (rAbs ≥ 1) and negative (rAbs < 1) result. A representative colorimetric results is showed upon each result *bar-red* positive result; *blue*/*purpl*e negative result.

First, we optimized the multiplex PCR amplification so that both two pathogens’ gene fragments are present with similar yields while maintaining PCR specificity. Primers were designed so as to produce amplicons showing similar sizes, since comparable hybridization efficiencies and profiles are desired [18]. The two *loci* were successfully amplified alone and then in a single multiplex reaction from DNA isolates of the pathogens (Additional file 1: Figure S1). Then, each Au-nanoprobe sequence was evaluated in terms of selectivity and specificity towards the respective target in solution (Fig. 2). Calibration of the Au-nanoprobe functionalization in presence of the respective target, but aggregated in its absence (see Additional file 1: Figures S3 and S4). No noteworthy loss in signal occurs when both pathogens amplicons are present in the mixture compared to each amplicon alone (Fig. 3).

**Fig. 4:**
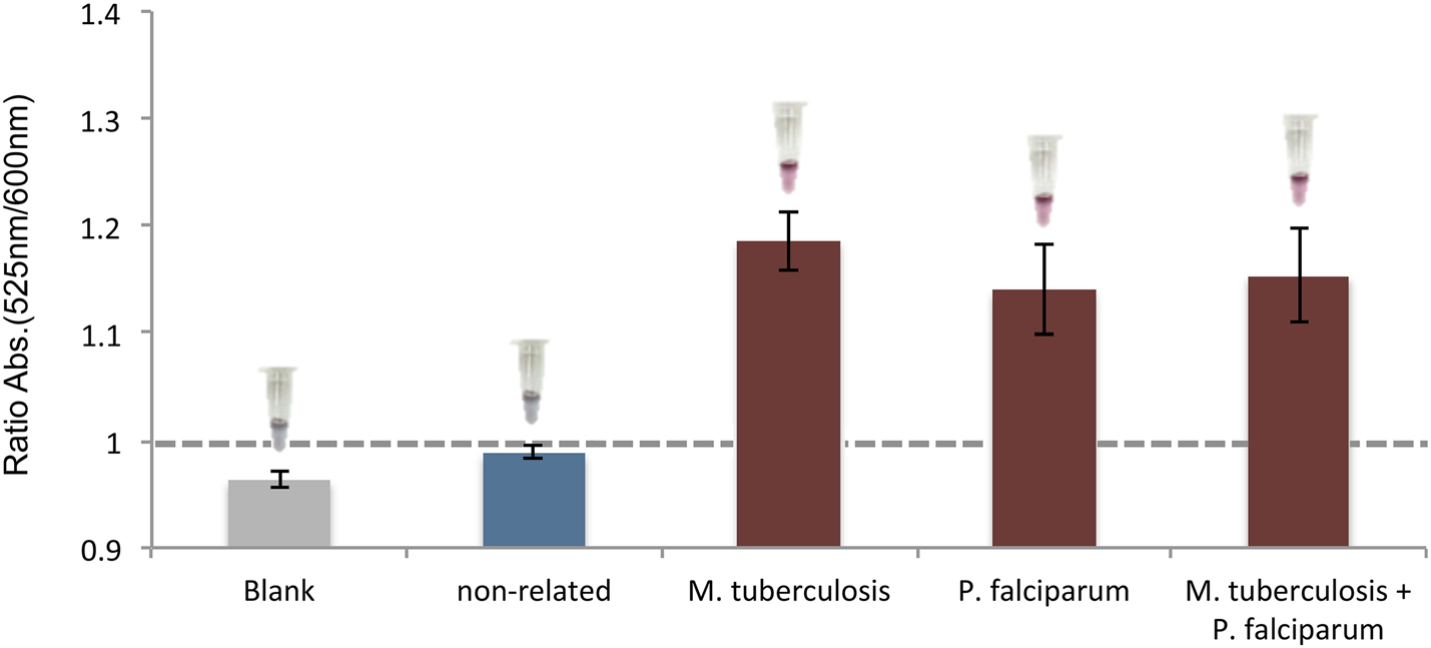
One probe, two pathogens assay. Use of a single Au-nanoprobe for sample characterization harbouring a single target (*M. tuberculosis* or *P. falciparum*) and both targets. The amount of total DNA in each sample is constant (60 µg/mL). Au-nanoprobes aggregation was measured by ratio of aggregation (ratio of SPR intensity at 525 and 600 nm) for the assay mixtures—2.5 nM Au-nanoprobe, 10 mM phosphate buffer (pH 8) + 0.1 M NaCl, purified dsDNA targets (PCR products) at a final concentration of 60 ng/µL. All spectrophotometric data was collected 30 min after salt addition and *error bars* represent the standard deviation of three independent assays. The *horizontal line* represents the threshold of 1 considered for discrimination between positive (rAbs ≥ 1) and negative (rAbs < 1) result. A representative colorimetric results is showed upon each result *bar-red* positive result; *blue*/*purple* negative result.

The principle of a multiple functionalized Au-nanoprobe relies on the simultaneous functionalization of AuNPs with specific oligonucleotides for both target sequences in a 1:80:80 (AuNP: oligo1: oligo2) ratio. This Au-nanoprobe was able to detect each target sequence individually in solution and when the target is in a multiplex PCR mixture (Figs. 3, 4).

## Conclusion

There is a continuous and strenuous demand for robust, yet simple molecular diagnostics capable to identify pathogens at point-of-care. The lack of laboratorial infrastructures in places at the forefront of disease areas, where simultaneous infections often occur, have been showing the relevance of developing simple assays that may provide immediate responses to the clinicians. Here, for the first time, the potential of using a single Au-nanoprobe for the specific detection of multiple targets in a single reaction is demonstrated. A two-step approach was designed based on the amplification of DNA samples followed by detection with a single Au-nanoprobe for the simultaneous specific detection of *M. tuberculosis* and *P. falciparum*, without loss of sensitivity. Due to its simplicity, this approach may easily be translated to remote areas. Nevertheless, under the present format, a thermocycler and spectrophotometer are still required but alternatively isothermal amplification [17] and cheap disposable platform [15] may eliminate the need of such need equipment with comparable results. The concept here presented may easily be extended to further pathogens and targets aiming at cost reduction and enlarge the range of molecular testing for pathogens at point of need.

## Additional file

Additional file 1:
**Table S1.** Multiplex PCR primers and Au-nanoprobes sequences.** Figure S1.** Electrophoretic analysis on 1% agarose gel: In lane 1 was used Thermo Scientific GeneRuler DNA Ladder Mix; Lane 2 and 3 Multiplex PCR products of *M. tuberculosis* and *P. falciparum*, respectively; Lane 4 Multiplex PCR product of *M. tuberculosis* and *P. falciparum*, Lane 5 negative control of the PCR reaction.** Figure S2.** Physical characterization of the synthesized gold nanoparticles. A) Transmission electron microscopy (TEM) imaging and inset size histogram frequency from ≈ 400 AuNPs counting; B) UV-Vis spectrum of spherical AuNPs, with a characteristic maximum absorption peak, C) Dynamic light scattering (DLS) measurements to define the hydrodynamic diameter of the AuNPs and zeta-potential (ζ-potential) as AuNP surface charge indicator. For DLS it was performed 3 runs per sample with 2000 measurements each and for ζ-potential a total of 5 runs per sample with 250 measurements.** Figure S3.** Aggregation profiles of the synthesized Au-nanoprobes. Aggregation measured as the ratio of localized surface plasmon resonance intensity at 525 nm and 600 nm for increasing salt concentrations (MgCl_2_). The minimum amount of salt required to cause aggregation was determined based on each Au-nanoprobe aggregation profiles. Ratio <1 was considered for full aggregation, 60 mM.** Figure S4.** Single pathogen Au-nanoprobe specificity analysis using synthetic targets. A 1 pmol concentration of synthetic targets (see Table S1) were individually tested with a MgCl_2_ concentration of 60 mM.

## Abbreviations

AuNPs: gold nanoparticles
LSPR: localized surface plasmon resonance
MDRTB: multi-drug resistant tuberculosis
MTBC: Mycobacterium tuberculosis complex
TB: tuberculosis
