# Supplementary material for: One nanoprobe, two pathogens: gold nanoprobes multiplexing for point-of-care
Source: J Nanobiotechnology. 2015 Aug 7;13:48. doi: 10.1186/s12951-015-0109-1 (PMC4527100; doi:10.1186/s12951-015-0109-1)
Supplement: Additional file 1: — Table S1. Multiplex PCR primers and Au-nanoprobes sequences. Figure S1. Electrophoretic analysis on 1% agarose gel: In lane 1 was used Thermo Scientific GeneRuler DNA Ladder Mix; Lane 2 and 3 Multiplex PCR products of M. tuberculosis and P. falciparum, respectively; Lane 4 Multiplex PCR product of M. tuberculosis and P. falciparum, Lane 5 negative control of the PCR reaction. Figure S2. Physical characterization of the synthesized gold nanoparticles. A) Transmission electron microscopy (TEM) imaging and inset size histogram frequency from ≈ 400 AuNPs counting; B) UV-Vis spectrum of spherical AuNPs, with a characteristic maximum absorption peak, C) Dynamic light scattering (DLS) measurements to define the hydrodynamic diameter of the AuNPs and zeta-potential (ζ-potential) as AuNP surface charge indicator. For DLS it was performed 3 runs per sample with 2000 measurements each and for ζ-potential a total of 5 runs per sample with 250 measurements. Figure S3. Aggregation profiles of the synthesized Au-nanoprobes. Aggregation measured as the ratio of localized surface plasmon resonance intensity at 525 nm and 600 nm for increasing salt concentrations (MgCl2). The minimum amount of salt required to cause aggregation was determined based on each Au-nanoprobe aggregation profiles. Ratio <1 was considered for full aggregation, 60 mM. Figure S4. Single pathogen Au-nanoprobe specificity analysis using synthetic targets. A 1 pmol concentration of synthetic targets (see Table S1) were individually tested with a MgCl2 concentration of 60 mM. [file 12951_2015_109_MOESM1_ESM.pdf]

Additional File 1

One nanoprobe, two pathogens: gold nanoprobes multiplexing for point-of-care

Bruno Veigas[a,b], Pedro Pedrosa[a], Fábio F. Carlos[a,c], Liliana Mancio-Silva[d], Ana Rita Grosso[d], Elvira Fortunato[b], Maria M. Mota[d], Pedro V. Baptista[a,*]

[a] CIGMH, UCIBIO, Departamento de Ciências da Vida, Faculdade de Ciências e Tecnologia, Universidade Nova de Lisboa, Caparica, Portugal

[b] CENIMAT/I3N, Departamento de Ciência dos Materiais, Faculdade de Ciências e Tecnologia, Universidade Nova de Lisboa, Caparica, Portugal

[c] STABVIDA, Investigação e Serviços em Ciências Biológicas, Lda. Madan Parque, 2825-182 Caparica, Portugal

[d] Instituto de Medicina Molecular, Universidade de Lisboa. Av. Prof. Egas Moniz, 1649-028, Lisboa, Portugal

[*] Corresponding Author: Nanomedicine@FCT, Departamento de Ciências da Vida, Faculdade de Ciências e Tecnologia, Universidade Nova de Lisboa, Campus de Caparica, 2829-516 Caparica, Portugal; Phone/Fax: +351 21 2948530; Email: pmvb@fct.unl.pt

Table S1 Multiplex PCR primers and Au-nanoprobes sequences

Name	Sequence
rpoB FW	5’ GAG AAT TCG GTC GGC GAG CTG ATC C 3’
rpoB RV	5’ CGA AGC TTG ACC CGC GCG TAC ACC 3’
Plasm FW	5’ CAGATGTCAGAGGTGAAATTC 3’
Plasm RV	5’ CATGCATCACCATCCAAGAAATCAA 3’
MTBC probe	Thiol - 5’ GAT CGC CTC CAC GTC C 3’
Plasmodium sp. Probe	Thiol - 5’ GGCGAGTATTCGCGCAAGCG 3’
MTBC Target	5’ TGGAACTATGAGTTGGACGTGGAGGCGATC 3’
Plasmodium sp. Target	5’ GTCAATTCTTTTAACTTTCTCGCTTGCGCGAATACTCGCC 3’
Non-related Target	5’ TTG AGT ATC AAG GTG ATC GCC TCC ACG TCC 3’

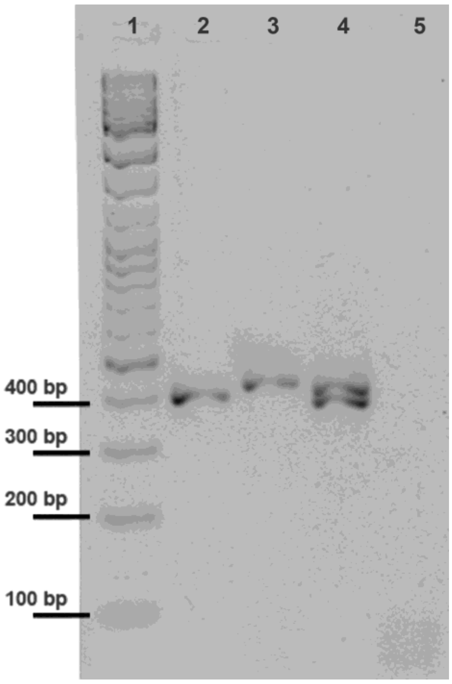

Fig. S1 Electrophoretic analysis on 1% agarose gel: In lane 1 was used Thermo Scientific GeneRuler DNA Ladder Mix; Lane 2 and 3 Multiplex PCR products of *M. tuberculosis* and *P. falciparum*, respectively; Lane 4 Multiplex PCR product of *M. tuberculosis* and *P. falciparum*, Lane 5 negative control of the PCR reaction.

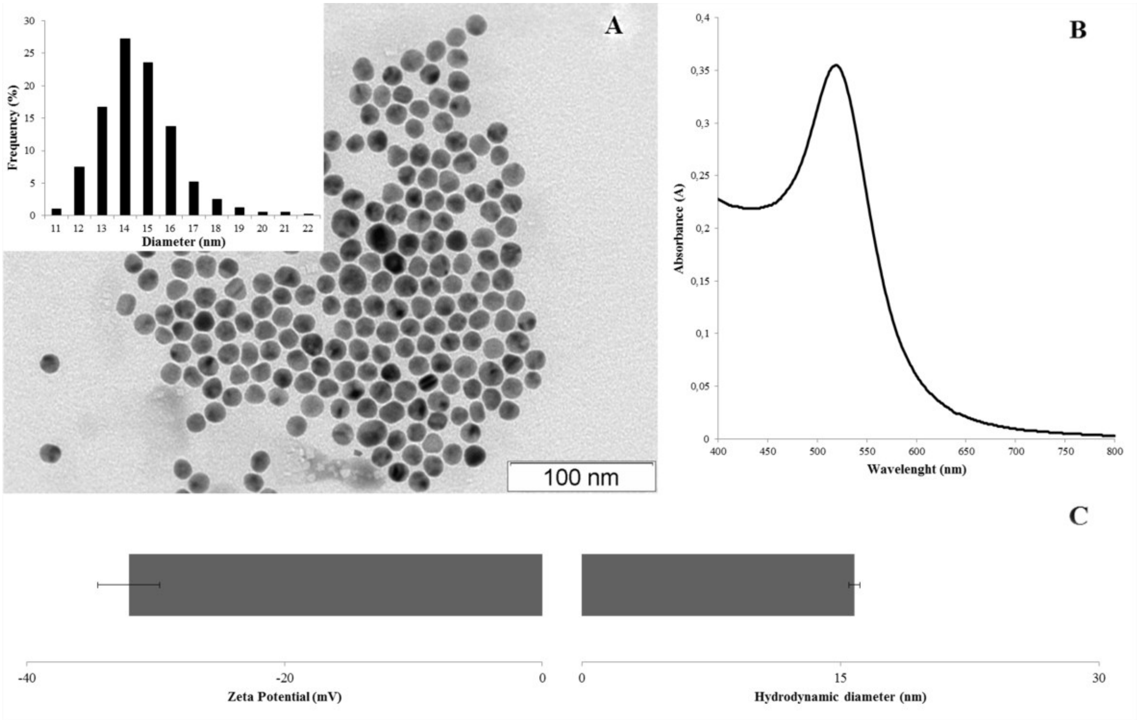

Fig. S2 Physical characterization of the synthesized gold nanoparticles. A) Transmission electron microscopy (TEM) imaging and inset size histogram frequency from ≈ 400 AuNPs counting; B) UV-Vis spectrum of spherical AuNPs, with a characteristic maximum absorption peak, C) Dynamic light scattering (DLS) measurements to define the hydrodynamic diameter of the AuNPs and zeta-potential (ζ-potential) as AuNP surface charge indicator. For DLS it was performed 3 runs per sample with 2000 measurements each and for ζ-potential a total of 5 runs per sample with 250 measurements.

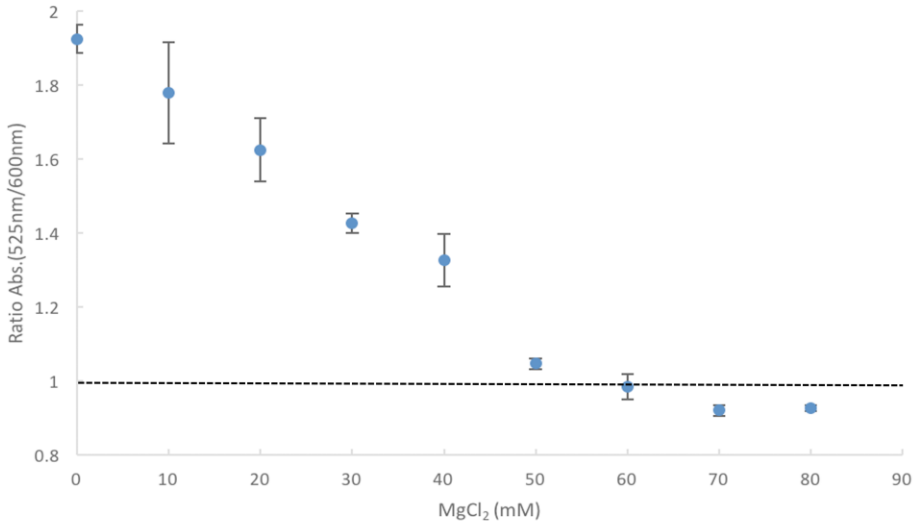

Fig. S3 Aggregation profiles of the synthesized Au-nanoprobes. Aggregation measured as the ratio of localized surface plasmon resonance intensity at 525 nm and 600 nm for increasing salt concentrations ($MgCl_2$). The minimum amount of salt required to cause aggregation was determined based on each Au-nanoprobe aggregation profiles. Ratio <1 was considered for full aggregation, 60mM.

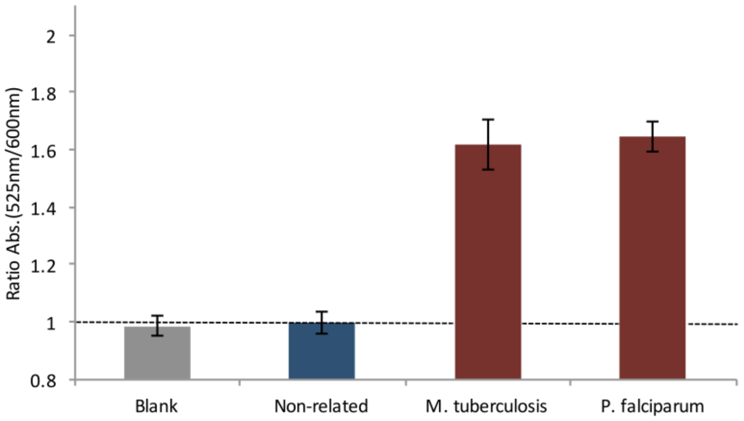

Fig. S4 Single pathogen Au-nanoprobe specificity analysis using synthetic targets. A 1 pmol concentration of synthetic targets (see Table S1) were individually tested with a $MgCl_2$ concentration of 60mM.
